# Predictive value of ibutilide response for postoperative recurrence following catheter ablation in persistent atrial fibrillation: a retrospective study

**DOI:** 10.3389/fcvm.2025.1623043

**Published:** 2025-08-06

**Authors:** Liuling Ma, Renkui Lai, Zhehan Guo, Tianjin Cai, Jingyi Huang, Jinxin Li

**Affiliations:** Department of Arrhythmia, The Second Affiliated Hospital of Guangzhou University of Chinese Medicine, Guangzhou, China

**Keywords:** atrial fibrillation, ibutilide, catheter ablation, recurrence, predictive factors, pulmonary vein isolation

## Abstract

**Objective:**

This study investigated efficacy of intraoperative ibutilide administration for cardioversion of persistent atrial fibrillation (AF) during catheter ablation and evaluated predictive factors for postoperative AF recurrence.

**Methods:**

A retrospective analysis was conducted involving 111 patients with persistent AF who underwent radiofrequency catheter ablation. Patients who failed to restore sinus rhythm after ablation received intravenous ibutilide (1–2 mg). Patients achieving sinus rhythm post-ibutilide administration were categorized as responders, while non-responders underwent electrical cardioversion. Clinical data were collected, and logistic regression was utilized to identify factors associated with ibutilide response and postoperative AF recurrence.

**Results:**

Sinus rhythm was restored by ibutilide in 73 patients (65.7%). Non-response to ibutilide was independently associated with longer AF duration (OR = 1.82), diabetes ellitus (OR = 2.27), coronary artery disease (OR = 2.56), increased ST2 (OR = 1.08), larger left atrial diameter (LAD) (OR = 1.25), elevated NT-proBNP (OR = 1.01), and higher CHA2DS2-VASc scores (OR = 1.96; all *P* < 0.05). AF recurrence within 3 months post-ablation was independently predicted by intraoperative ibutilide non-response (OR = 5.317), older age (OR = 1.213), diabetes mellitus (OR = 0.14), increased LAD (OR = 1.211), elevated ST2 (OR = 1.139), elevated hs-CRP (OR = 1.276), and higher CHA_2_DS_2_-VASc scores (OR = 2.736; all *P* < 0.05).

**Conclusion:**

Intraoperative ibutilide responsiveness significantly predicts postoperative AF recurrence in patients undergoing catheter ablation for persistent AF. Assessing ibutilide response may enhance risk stratification and guide personalized treatment strategies.

## Introduction

Atrial fibrillation (AF) is among the most prevalent cardiac arrhythmias encountered clinically, characterized by disorganized atrial electrical activity and compromised atrial function. While its prevalence is low (<1%) in younger individuals, the incidence increases significantly with advancing age, posing substantial risks of ischemic stroke, heart failure, and increased morbidity and mortality (1, 2). Pulmonary vein isolation (PVI) via radiofrequency catheter ablation (RFCA) has emerged as a cornerstone intervention in AF management, given that pulmonary veins are implicated as primary AF trigger sites in more than 90% of cases (3). Nevertheless, successful rhythm termination through isolated PVI remains limited, particularly in persistent AF, with relatively high recurrence rates reported post-ablation. Consequently, repeated interventions may become necessary for many patients, underscoring the importance of identifying factors predictive of AF recurrence to guide clinical decisions and enhance long-term therapeutic efficacy (4).

Adjunctive use of antiarrhythmic agents during catheter ablation has become common practice to facilitate restoration of sinus rhythm. Ibutilide, a novel class III antiarrhythmic drug, acts primarily by blocking rapid outward potassium currents, resulting in prolonged atrial refractoriness and increased likelihood of AF termination (5). Previous research has indicated cardioversion success rates ranging between 50.5% and 56% with ibutilide administration in patients who had not undergone ablation (6, 7). Importantly, after achieving PVI, the interruption of electrical conduction between pulmonary veins and the left atrium appears to further enhance the cardioversion efficacy of ibutilide, with studies reporting conversion rates up to approximately 62.3% (8).

Despite advancements in understanding AF pathophysiology, definitive consensus on the predictors of AF recurrence post-ablation remains elusive. Several previous studies have highlighted various factors potentially associated with recurrence risk. For example, Liu et al. proposed that responsiveness to ibutilide could serve as an early indicator of subsequent AF recurrence (9). Additionally, studies have identified age, enlarged left atrial diameter (LAD), atrial fibrosis, and serum biomarkers such as NT-proBNP as significant predictors of recurrence (10, 11). Elevated preoperative soluble ST2 (sST2) concentrations have also shown promising predictive value, with reported sensitivities and specificities of approximately 77.3% and 79.5%, respectively (12). However, due to variability among study populations and methodological differences, reliable clinical predictors remain inadequately defined.

Therefore, this retrospective study aimed to evaluate the predictive value of intraoperative ibutilide response and associated clinical and laboratory parameters on the recurrence of AF following catheter ablation. Identification of robust predictors could facilitate early risk stratification and tailored management strategies in patients with persistent AF undergoing RFCA.

## Methods

### Study design

This retrospective observational study included 111 consecutive patients with persistent AF who underwent RFCA at Guangdong Provincial Hospital of Traditional Chinese Medicine between January 2019 and May 2023. This study was approved by Ethics Committee of The Second Affiliated Hospital of Guangzhou University of Chinese Medicine. Written informed consent was obtained from all patients prior to the procedure. The study was conducted in accordance with the ethical principles outlined in the Declaration of Helsinki. All participants had persistent AF, defined according to the criteria specified in Atrial Fibrillation: Current Understanding and Treatment Recommendations—2021 (13). Patients who failed to achieve sinus rhythm following the initial ablation procedure were enrolled for further analysis.

### Inclusion and exclusion criteria

Patients were eligible for inclusion based following criteria: (1) diagnosis of persistent AF according to current guidelines; (2) absence of contraindications for RFCA; (3) inadequate response or intolerance to pharmacological therapy, or refusal of antiarrhythmic medications; and (4) age less than 85 years.

Patients were excluded according to following conditions: (1) severe heart failure [New York Heart Association (NYHA) class ≥III]; (2) history of acute myocardial infarction, cardiomyopathy, rheumatic valvular heart disease, or cardiac surgical interventions (including valve replacement, coronary artery bypass grafting, or coronary interventions) within the previous 3 months; (3) severe hepatic or renal dysfunction; or (4) presence of malignant tumors, significant infections, or hematologic disorders.

### Data collection

Baseline clinical and demographic data were retrospectively retrieved from electronic medical records, including patient age, sex, body mass index (BMI), and medical history (hypertension, diabetes mellitus, cerebral infarction, coronary artery disease, smoking status, and alcohol consumption). Laboratory parameters recorded at admission included serum creatinine (sCr), fasting glucose (Glu), hemoglobin A1c (HbA1c), lipid profiles comprising total cholesterol (TC), low-density lipoprotein cholesterol (LDL-C), high-density lipoprotein cholesterol (HDL-C), triglycerides (TG), as well as biomarkers including high-sensitivity C-reactive protein (hs-CRP), N-terminal pro-brain natriuretic peptide (NT-proBNP), and soluble growth stimulation expressed factor 2 (ST2). Echocardiographic measurements collected were left ventricular ejection fraction (LVEF), left atrial diameter (LAD), left ventricular end-diastolic diameter (LVEDD), and left ventricular end-systolic diameter (LVESD).

### CHA_2_DS_2_-VASc scoring

CHA_2_DS_2_-VASc scores were calculated to assess stroke risk based on the following clinical characteristics: recent congestive heart failure (1 point), hypertension (1 point), age ≥75 years (2 points), diabetes mellitus (1 point), prior thromboembolic events (stroke, transient ischemic attack, or systemic embolism; 2 points), vascular disease (including prior myocardial infarction, peripheral artery disease, or aortic plaque; 1 point), age between 65 and 74 years (1 point), and female sex (1 point), with a maximum score of 9 points.

### Catheter ablation procedure

All ablation procedures were performed under three-dimensional electroanatomic mapping guidance. PVI was routinely performed, ensuring bidirectional conduction block at the pulmonary vein ostia. Additional linear ablations were completed on the left atrial roof and posterior wall until bidirectional linear conduction block was documented. Patients who did not revert to sinus rhythm within 20 min post-ablation received intravenous ibutilide administration (1 mg diluted in normal saline to a concentration of 0.05 mg/ml, infused over 10 min via the femoral vein). If sinus rhythm was not restored within 20 min following the initial dose, a second dose was administered with a maximum cumulative dose of 2 mg. Patients converting to sinus rhythm were defined as the ibutilide-effective group. Those who continued with AF or developed atrial flutter despite ibutilide administration subsequently underwent synchronized direct-current cardioversion at 200 J, constituting the ibutilide-ineffective group. Following rhythm conversion, electroanatomic mapping was repeated to confirm complete pulmonary vein isolation, bidirectional conduction block, and any required additional substrate modification guided by mapping findings.

### Follow-up

All patients underwent routine outpatient follow-up visits at 1 and 3 months post-procedure. Follow-up assessments involved standard 12-lead ECG and 24-h Holter monitoring. AF recurrence was defined as the detection of any atrial tachyarrhythmia lasting at least 30 s on ECG or Holter recordings. AF recurrence rates were subsequently calculated.

### Statistical analysis

Statistical analyses were conducted using SPSS software version 26.0 (IBM Corp., Armonk, NY, USA). Continuous variables were presented as mean ± standard deviation (SD), while categorical variables were expressed as numbers and percentages. Between-group comparisons were performed using independent samples *t*-tests or Mann–Whitney *U* tests for continuous variables, and Chi-square tests or Fisher's exact tests for categorical variables. Univariate and multivariate logistic regression analyses were conducted to identify independent predictors of intraoperative ibutilide response and postoperative AF recurrence, with results presented as odds ratios (OR) and corresponding 95% confidence intervals (CI). Statistical significance was set at a two-sided *P*-value <0.05.

## Results

### Clinical characteristics

A total of 111 patients (60 males and 51 females) with persistent AF who underwent radiofrequency catheter ablation were included in this retrospective analysis. Mean age was 64.96 ± 10.65 years, and average AF duration prior to intervention was 3.17 ± 1.72 years. Echocardiographic evaluation revealed an average left atrial diameter (LAD) of 42.90 ± 3.87 mm and a mean LVEF of 65.77% ± 6.20%. Intraoperatively, intravenous ibutilide administration successfully restored sinus rhythm in 73 patients (65.7%), whereas 38 patients (34.3%) did not achieve sinus rhythm conversion, including 4 patients who converted to atrial flutter. Among 111 patients who received ibutilide after radiofrequency ablation, during the subsequent 6-h monitoring period no malignant arrhythmias, such as polymorphic ventricular tachycardia or torsades de pointes occurred. However, one patient developed third-degree atrioventricular block immediately after the ibutilide bolus; a temporary pacemaker was placed intraoperatively and was successfully removed approximately six hours later once sinus rhythm had been restored.

During the 3-month follow-up period, AF recurrence was observed in 37 patients (33.3%) (Table 1). Within 3 months postoperatively, all patients received anticoagulation therapy with rivaroxaban. For antiarrhythmic treatment after surgery, every patient was maintained on extended-release metoprolol succinate for 3 months, and 36 of these patients also received amiodarone concurrently for the same duration.

**Table 1:** Baseline clinical characteristics of patients with and without AF recurrence.

Parameters	Recurrence (*n* = 37)	Non-recurrence (*n* = 74)	*Z*/*X*^2^/*t*	*P*
Age (years old)	69 (82.6, 71)	54 (74.25, 63.5)	−5.656	<0.001
Gender			1.469	0.225
Male	17 (45.95)	43 (58.11)		
Female	20 (54.05)	31 (41.89)		
Smoking [*n* (%)]	16 (43.24)	26 (35.14)	0.689	0.406
Alcohol consumption history [*n* (%)]	9 (24.32)	12 (16.22)	1.057	0.304
Height (cm)	162.03 ± 9.11	162.97 ± 7.91	−0.565	0.573
Body weight (kg)	68.54 ± 9.4	69.3 ± 9.52	−0.397	0.692
BMI (kg/m^2^)	23.91 (32.00, 25.97)	23.71 (32.89, 26.00)	−0.141	0.888
Hypertension [*n* (%)]	36 (97.3)	64 (86.49)	3.229	0.072
Diabetes [*n* (%)]	20 (54.05)	16 (21.62)	11.84	0.001
Coronary artery disease [*n* (%)]	18 (48.65)	19 (25.68)	5.858	0.016
Rheumatic heart disease [*n* (%)]	0 (0)	1 (1.35)	0.505	0.478
Cerebrovascular disease [*n* (%)]	3 (8.11)	4 (5.41)	0.305	0.581
Heart failure [*n* (%)]	4 (10.81)	5 (6.76)	0.544	0.461
Duration of atrial fibrillation (years)	3 (7.1, 3)	1 (6, 3)	−2.485	0.013
Successful cardioversion with ibutilide [*n* (%)				
Yes	15 (40.54)	58 (78.38)	15.686	<0.001
No	22 (59.46)	16 (21.62)		
CHA_2_DS_2_-VASc score	3 (6.1, 4)	1 (4.25, 2)	−5.327	<0.001
hs-CRP (mg/L)	4.5 (1.98, 6.8)	3.53 (1.42, 4.93)	−2.365	0.018
NT- proBNP (ng/L)	554 (364.5, 827.05)	381.5 (177, 636.25)	−2.524	0.012
ST2	15 (10, 21)	10 (7, 14)	−3.943	<0.001
sCr (µmol/L)	74 (63, 87)	68.5 (56, 80)	−1.785	0.074
GLU (mmol/L)	5.7 (5.3, 6.9)	5.16 (4.88, 5.9)	−2.875	0.004
TC (mmol/L)	4.52 (3.9, 6.2)	4.85 (4.11, 5.49)	−0.357	0.721
TG (mmol/L)	1.6 (1.32, 2.12)	1.7 (1.35, 2.11)	−0.122	0.903
LDL-C (mmol/L)	3.1 (2.28, 3.61)	3.05 (2.52, 3.7)	−0.041	0.968
HDL-C (mmol/L)	0.92 ± 0.18	0.96 ± 0.14	−1.088	0.281
HbA1c (%)	5.8 (5.3, 6.85)	5.4 (5.1, 5.9)	−2.932	0.003
LAD (mm)	44.59 ± 3.71	42.05 ± 3.69	3.412	0.001
LVEF (%)	65 (60, 68)	68 (65, 70)	−3.232	0.001
LVEDD (mm)	45 (43, 47)	44 (43, 46)	−1.116	0.264
LVED (mm)	26 (23, 29)	25 (21, 27)	−1.512	0.131

Continuous variables presented as mean ± standard deviation or median (Q3, Q1). Categorical variables presented as number (percentage). AF, atrial fibrillation; BMI, body mass index; hs-CRP, high-sensitivity C-reactive protein; NT-proBNP, N-terminal pro-brain natriuretic peptide; ST2, soluble growth stimulation expressed factor 2; sCr, serum creatinine; GLU, glucose; TC, total cholesterol; TG, triglycerides; LDL-C, low-density lipoprotein cholesterol; HDL-C, high-density lipoprotein cholesterol; LAD, left atrial diameter; LVEF, left ventricular ejection fraction; LVEDD, left ventricular end-diastolic diameter; LVESD, left ventricular end-systolic diameter.

### Predictive factors for intraoperative ibutilide cardioversion response

Patients who failed to respond to ibutilide intraoperatively had significantly longer durations of AF compared to those with successful cardioversion. Multivariate logistic regression analysis identified longer AF duration as an independent predictor of intraoperative non-response to ibutilide (Table 2). Logistic regression using intraoperative non-response to ibutilide as the dependent variable demonstrated significant associations between non-response to ibutilide and longer AF duration [OR = 1.82, 95% CI (1.35–2.35), *P* < 0.05], diabetes mellitus [OR = 2.27, 95% CI (1.01–5.26), *P* < 0.05], coronary artery disease [OR = 2.56, 95% CI (1.12–5.81), *P* < 0.05], elevated ST2 levels [OR = 1.08, 95% CI (1.01–1.13), *P* < 0.05], increased LAD [OR = 1.25, 95% CI (1.11–1.42), *P* < 0.05], elevated NT-proBNP [OR = 1, 95% CI (1.000–1.002), *P* < 0.05], and higher CHA2DS2-VASc scores [OR = 1.96, 95% CI (1.38–2.80), *P* < 0.05] (Table 2).

**Table 2:** Multivariate logistic regression of factors predicting non-response to ibutilide.

Variables	OR	95% CI	*P*-value
Duration of AF (years)	1.82	1.35–2.35	<0.001
Diabetes mellitus	2.27	1.01–5.26	0.048
Coronary artery disease	2.56	1.12–5.81	0.025
CHA₂DS₂-VASc score	1.96	1.38–2.80	<0.001
NT-proBNP (ng/L)	1.01	1.000–1.002	0.005
ST2	1.08	1.01–1.13	0.014
LAD (mm)	1.25	1.11–1.42	<0.001

OR, odds ratio; CI, confidence interval; AF, atrial fibrillation; NT-proBNP, N-terminal pro-brain natriuretic peptide; ST2, soluble growth stimulation expressed factor 2; LAD, left atrial diameter.

### Predictive factors for AF recurrence after catheter ablation

Logistic regression analysis was performed with postoperative AF recurrence as the dependent variable, including statistically significant variables identified in univariate analyses as independent variables. Multivariate analysis revealed that older age [OR = 1.213, 95% CI (1.061–1.386), *P* < 0.05], diabetes mellitus [OR = 0.140, 95% CI (0.037–0.528), *P* < 0.05], elevated hs-CRP [OR = 1.276, 95% CI (1.031–1.580), *P* < 0.05], increased soluble ST2 [OR = 1.139, 95% CI (1.036–1.252), *P* < 0.05], larger LAD [OR = 1.211, 95% CI (1.015–1.446), *P* < 0.05], higher CHA2DS2-VASc scores [OR = 2.736, 95% CI (1.704–4.394), *P* < 0.05], and intraoperative ibutilide non-response [OR = 5.317, 95% CI (1.932–14.628), *P* < 0.05] were independently associated with a higher risk of AF recurrence after catheter ablation (Table 3).

**Table 3:** Multivariate logistic regression analysis of predictive factors for AF recurrence after catheter ablation.

Variables	OR	95% CI	*P*-value
Age (years old)	1.213	1.104–1.333	<0.001
Diabetes mellitus	0.14	0.041–0.474	0.002
hs-CRP (mg/L)	1.276	1.002–1.626	0.048
NT-proBNP (ng/L)	0.999	0.998–1.000	0.056
LVEF (%)	0.925	0.842–1.016	0.103
Intraoperative ibutilide non-response	5.317	2.253–12.545	<0.001
ST2	1.139	1.059–1.224	<0.001
LAD (mm)	1.211	1.075–1.365	0.002
CHA₂DS₂-VASc score	2.736	1.759–4.254	<0.001

AF, atrial fibrillation; OR, odds ratio; CI, confidence interval; CRP, C-reactive protein; LVEF, left ventricular ejection fraction; ST2, soluble growth stimulation expressed factor 2; LAD, left atrial diameter.

## Discussion

AF is one of the most frequently encountered cardiac arrhythmias in clinical practice, characterized by disrupted atrial electrical activity and impaired mechanical atrial function. The rising incidence of AF has become a significant public health concern due to associated severe complications such as ischemic stroke, heart failure, and increased morbidity and mortality. RFCA, particularly pulmonary vein isolation PVI, represents a well-established therapeutic strategy for persistent AF. However, despite advances in ablation techniques, recurrence rates post-ablation remain high, reflecting the complexity of AF pathophysiology and interpatient variability. Therefore, identifying reliable preprocedural or intraprocedural factors predictive of AF recurrence remains clinically relevant for risk stratification and targeted management. In our retrospective cohort of patients with persistent AF undergoing PVI, ibutilide successfully restored sinus rhythm in approximately two-thirds of cases. Notably, longer pre-procedural AF duration independently predicted failure of acute drug-induced cardioversion. Furthermore, intraoperative non-response to ibutilide, as well as older age, the presence of diabetes, larger left atrial diameter, higher serum sST2 and hs-CRP levels, and elevated CHA₂DS₂-VASc scores, each independently portended an increased risk of AF recurrence following catheter ablation.

Ibutilide, a class III antiarrhythmic agent, promotes cardioversion through prolongation of atrial refractoriness and QT intervals, thereby facilitating termination of AF or atrial flutter (14). In the current cohort, ibutilide administration intraoperatively resulted in a sinus rhythm conversion rate of approximately 65.7%. This finding aligns with previously reported success rates of ibutilide (approximately 62.3%) in patients undergoing RFCA (8). Although direct-current cardioversion typically demonstrates higher efficacy (∼85%), ibutilide offers practical advantages by eliminating the need for patient sedation and avoiding disruptions associated with electrical cardioversion, such as displacement of mapping patches (15, 16).

Our analysis further demonstrated that longer AF duration significantly predicted non-response to intraoperative ibutilide administration. In multivariate analysis, additional factors such as diabetes mellitus, coronary artery disease, elevated ST2, increased LAD, elevated NT-proBNP, and higher CHA_2_DS_2_-VASc scores were significantly associated with ibutilide non-response. These results suggest that structural and functional atrial remodeling, reflected by these clinical parameters, may directly influence susceptibility to antiarrhythmic pharmacologic intervention during catheter ablation.

Moreover, intraoperative ibutilide non-response emerged as a robust independent predictor of AF recurrence post-ablation, consistent with previous studies suggesting that responsiveness to pharmacological cardioversion may reflect the extent of underlying atrial remodeling and predict subsequent clinical outcomes (9). Additionally, we observed significant correlations between AF recurrence and clinical factors including advanced age, diabetes mellitus, larger LAD, higher serum ST2 and hs-CRP levels, and increased CHA_2_DS_2_-VASc scores. Notably, LAD, ST2, diabetes mellitus, and CHA_2_DS_2_-VASc scores predicted both ibutilide non-response and AF recurrence, indicating shared pathophysiological pathways involving structural and inflammatory remodeling of the atrium.

Enlarged LAD is a recognized marker of atrial structural remodeling, fibrosis, increased atrial pressures, and compromised mechanical function, which collectively promote recurrence post-ablation (17, 18). Prior studies have similarly demonstrated that larger LAD independently predicts short-term AF recurrence after RFCA (17). In our cohort, elevated serum sST2 levels were independently predictive of recurrence, likely reflecting ongoing inflammation and fibrotic remodeling within atrial tissues. Consistent with earlier research, elevated sST2 concentrations have been identified as sensitive biomarkers of AF recurrence risk following ablation, due to their association with enhanced inflammatory and oxidative stress responses (19, 20). Elevated hs-CRP, another inflammatory marker, was likewise independently predictive of AF recurrence, highlighting inflammation as a key contributor to post-ablation arrhythmia recurrence.

Furthermore, the CHA_2_DS_2_-VASc scoring system, primarily established for thromboembolic risk stratification, has previously been correlated with increased recurrence risk after AF ablation procedures (21). The present study provides additional support for CHA_2_DS_2_-VASc as a practical and readily applicable clinical tool for early identification of patients at elevated recurrence risk, potentially guiding more individualized patient management strategies.

Collectively, our findings underscore the clinical utility of assessing intraoperative ibutilide responsiveness and key clinical indicators such as age, LAD, diabetes, inflammatory biomarkers, and CHA_2_DS_2_-VASc scores for predicting AF recurrence risk. Recognizing patients at high risk of recurrence allows for tailored postoperative management, closer monitoring, and possibly intensified antiarrhythmic or anticoagulant therapy.

Several limitations of this retrospective study merit discussion. First, as a single-center retrospective analysis, potential selection biases and limited generalizability of findings may exist. Second, the administration of ibutilide intraoperatively could have influenced clinical decisions regarding additional substrate ablation or postoperative management, indirectly affecting outcomes. Asymptomatic AF recurrences may have been underestimated during follow-up due to reliance on scheduled outpatient ECG and Holter monitoring rather than continuous rhythm monitoring. Future prospective studies using implantable cardiac rhythm monitors would provide more accurate recurrence assessments and validation of predictive biomarkers. Finally, although endocardial scar burden assessed via electroanatomical mapping has emerged as a powerful predictor of AF recurrence (22), we did not quantify scar burden in this cohort. Incorporating scar assessment into prospective investigations could determine whether it outperforms clinical AF duration in predicting both ibutilide response and long-term ablation success.

## Conclusion

In patients with persistent AF undergoing radiofrequency catheter ablation, intraoperative cardioversion response to ibutilide was independently predictive of postoperative AF recurrence. Additional significant predictors identified included advanced age, diabetes mellitus, increased left atrial diameter, elevated serum ST2, hs-CRP levels, and higher CHA_2_DS_2_-VASc scores. These clinical and biochemical markers may serve as valuable tools for preprocedural risk stratification, guiding individualized patient management, follow-up strategies, and therapeutic decision-making aimed at reducing recurrence rates following catheter ablation.

## Data Availability

The original contributions presented in the study are included in the article/Supplementary Material, further inquiries can be directed to the corresponding author.

## References

[1] Brundel BJJM Ai X Hills MT Kuipers MF Lip GYH de Groot NMS. Atrial fibrillation. Nat Rev Dis Primers. (2022) 8(1):21. 10.1038/s41572-022-00347-9 35393446

[2] Lippi G Sanchis-Gomar F Cervellin G. Global epidemiology of atrial fibrillation: an increasing epidemic and public health challenge. Int J Stroke. (2021) 16(2):217–21. 10.1177/1747493019897870 31955707

[3] Haïssaguerre M Jaïs P Shah DC Takahashi A Hocini M Quiniou G Spontaneous initiation of atrial fibrillation by ectopic beats originating in the pulmonary veins. N Engl J Med. (1998) 339(10):659–66. 10.1056/NEJM199809033391003 9725923

[4] Imberti JF Ding WY Kotalczyk A Zhang J Boriani G Lip G Catheter ablation as first-line treatment for paroxysmal atrial fibrillation: a systematic review and meta-analysis. Heart. (2021) 107(20):1630–6. 10.1136/heartjnl-2021-319496 34261737

[5] Burashnikov A Di Diego JM Patocskai B Echt DS Belardinelli L Antzelevitch C. Effect of flecainide and ibutilide alone and in combination to terminate and prevent recurrence of atrial fibrillation. Circ Arrhythm Electrophysiol. (2024) 17(1):e012454. 10.1161/CIRCEP.123.012454 38146652 PMC10793769

[6] Vinson DR Lugovskaya N Warton EM Rome AM Stevenson MD Reed ME Ibutilide effectiveness and safety in the cardioversion of atrial fibrillation and flutter in the community emergency department. Ann Emerg Med. (2018) 71(1):96–108.e2. 10.1016/j.annemergmed.2017.07.481 28969929

[7] deSouza IS Tadrous M Sexton T Benabbas R Carmelli G Sinert R. Pharmacologic cardioversion of recent-onset atrial fibrillation and flutter in the emergency department: a systematic review and network meta-analysis. Ann Emerg Med. (2020) 76(1):14–30. 10.1016/j.annemergmed.2020.01.013 32173135

[8] Wu Y Gao P Liu Y Fang Q. Response to ibutilide and the long-term outcome after catheter ablation for non-paroxysmal atrial fibrillation. Cardiovasc J Afr. (2022) 33(3):112–6. 10.5830/CVJA-2021-044 34704587 PMC9540325

[9] Liu X He Y Gui C Wen W Jiang Z Zhong G Comparison of clinical outcomes of ibutilide-guided cardioversion and direct current synchronized cardioversion after radiofrequency ablation of persistent atrial fibrillation. Front Cardiovasc Med. (2023) 10:1141698. 10.3389/fcvm.2023.1141698 38028483 PMC10658000

[10] Kranert M Shchetynska-Marinova T Liebe V Doesch C Papavassiliu T Akin I Recurrence of atrial fibrillation in dependence of left atrial volume index. In Vivo. (2020) 34(2):889–96. 10.21873/invivo.11854 32111800 PMC7157897

[11] Xu X Tang Y. Relationship between brain natriuretic peptide and recurrence of atrial fibrillation after successful electrical cardioversion: an updated meta-analysis. Braz J Cardiovasc Surg. (2017) 32(6):530–5. 10.21470/1678-9741-2017-0008 29267617 PMC5731309

[12] Okar S Kaypakli O Şahin DY Koç M. Fibrosis marker soluble ST2 predicts atrial fibrillation recurrence after cryoballoon catheter ablation of nonvalvular paroxysmal atrial fibrillation. Korean Circ J. (2018) 48(10):920–9. 10.4070/kcj.2018.0047 30238709 PMC6158454

[13] Cheung CC Nattel S Macle L Andrade JG. Management of atrial fibrillation in 2021: an updated comparison of the current CCS/CHRS, ESC, and AHA/ACC/HRS guidelines. Can J Cardiol. (2021) 37(10):1607–18. 10.1016/j.cjca.2021.06.011 34186113

[14] Rogers KC Wolfe DA. Ibutilide: a class III rapidly acting antidysrhythmic for atrial fibrillation or atrial flutter. J Emerg Med. (2001) 20(1):67–71. 10.1016/S0736-4679(00)00274-2 11165840

[15] Pisters R Nieuwlaat R Prins MH Le Heuzey JY Maggioni AP Camm AJ Clinical correlates of immediate success and outcome at 1-year follow-up of real-world cardioversion of atrial fibrillation: the euro heart survey. EP Europace. (2012) 14(5):666–74. 10.1093/europace/eur406 22223715

[16] Crijns HJGM Weijs B Fairley AM Lewalter T Maggioni AP Martín A Contemporary real life cardioversion of atrial fibrillation: results from the multinational RHYTHM-AF study. Int J Cardiol. (2014) 172(3):588–94. 10.1016/j.ijcard.2014.01.099 24556445

[17] Istratoaie S Vesa ȘC Cismaru G Pop D Roșu R Puiu M Value of left atrial appendage function measured by transesophageal echocardiography for prediction of atrial fibrillation recurrence after radiofrequency catheter ablation. Diagnostics (Basel). (2021) 11(8):1465. 10.3390/diagnostics11081465 34441399 PMC8394000

[18] Baek YS Choi JI Kim YG Lee KN Roh SY Ahn J Atrial substrate underlies the recurrence after catheter ablation in patients with atrial fibrillation. J Clin Med. (2020) 9(10):3164. 10.3390/jcm9103164 33007810 PMC7601892

[19] Budzianowski J Hiczkiewicz J Burchardt P Pieszko K Rzeźniczak J Budzianowski P Predictors of atrial fibrillation early recurrence following cryoballoon ablation of pulmonary veins using statistical assessment and machine learning algorithms. Heart Vessels. (2019) 34(2):352–9. 10.1007/s00380-018-1244-z 30140958 PMC6510876

[20] Fan J Li Y Yan Q Wu W Xu P Liu L Higher serum sST2 is associated with increased left atrial low-voltage areas and atrial fibrillation recurrence in patients undergoing radiofrequency ablation. J Interv Card Electrophysiol. (2022) 64(3):733–42. 10.1007/s10840-022-01153-9 35175491

[21] Vitali F Serenelli M Airaksinen J Pavasini R Tomaszuk-Kazberuk A Mlodawska E CHA2DS2-VASc score predicts atrial fibrillation recurrence after cardioversion: systematic review and individual patient pooled meta-analysis. Clin Cardiol. (2019) 42(3):358–64. 10.1002/clc.23147 30597581 PMC6712331

[22] Brunner G Abboud L Shaikh KA Dave AS Morrisett J Zoghbi WA Left atrial scar burden determined by delayed enhancement cardiac magnetic resonance at post radiofrequency ablation: association with atrial fibrillation recurrence. J Cardiovasc Magn Reson. (2012) 14(Suppl 1):P204. 10.1186/1532-429X-14-S1-P204

